# Integrated platform for EV separation and controlled release based on gelatin microspheres for diabetic wound treatment

**DOI:** 10.1093/rb/rbag043

**Published:** 2026-03-07

**Authors:** Yiqing Zhang, Qilin Huang, Hao Meng, Junli Chen, Yaying Hao, Xi Liu, Yangmengyuan Xu, Liqian Ma, Zhan Xu, Kui Ma, Wenzhi Hu, Xiaohua Pan, Xiaobing Fu, Cuiping Zhang

**Affiliations:** Medical Innovation Research Department, PLA General Hospital and PLA Medical College, Beijing 100048, China; Medical Innovation Research Department, PLA General Hospital and PLA Medical College, Beijing 100048, China; Medical Innovation Research Department, PLA General Hospital and PLA Medical College, Beijing 100048, China; Medical Innovation Research Department, PLA General Hospital and PLA Medical College, Beijing 100048, China; Medical Innovation Research Department, PLA General Hospital and PLA Medical College, Beijing 100048, China; Medical Innovation Research Department, PLA General Hospital and PLA Medical College, Beijing 100048, China; Medical Innovation Research Department, PLA General Hospital and PLA Medical College, Beijing 100048, China; Medical Innovation Research Department, PLA General Hospital and PLA Medical College, Beijing 100048, China; Medical Innovation Research Department, PLA General Hospital and PLA Medical College, Beijing 100048, China; Medical Innovation Research Department, PLA General Hospital and PLA Medical College, Beijing 100048, China; Medical Innovation Research Department, PLA General Hospital and PLA Medical College, Beijing 100048, China; Institute of Clinical Translation and Regenerative Medicine, The Second Affiliated Hospital of Shenzhen University, Shenzhen 518100, China; Medical Innovation Research Department, PLA General Hospital and PLA Medical College, Beijing 100048, China; Medical Innovation Research Department, PLA General Hospital and PLA Medical College, Beijing 100048, China

**Keywords:** extracellular vesicles, gelatin-microsphere, diabetic wound healing

## Abstract

Diabetic wounds exhibit impaired healing owing to multiple pathological factors and current treatment options remain limited, underscoring the urgent need for novel therapeutic strategies. Extracellular vesicles (EVs) derived from mesenchymal stem cells (MSCs) are emerging as promising candidates for wound healing. However, the process from isolation to application of EVs is complex and time-consuming, which probably influences the activity and the therapeutic effects of EVs. To address these issues, we created the integrated platform for EV separation and controlled release. Gelatin microspheres (GMS) were prepared and functionalized with the variable domain of heavy-chain-only antibodies (GMS-VHH). GMS-VHH enabled the efficient capture of CD9-positive EVs in the culture supernatant of MSCs, forming EVs-loaded gelatin microspheres (GMS@EVs). The characterization of EVs demonstrated that the GMS-VHH isolation method outperformed traditional ultracentrifugation in terms of EV structural integrity. GMS@EVs exhibited excellent biocompatibility and promoted the proliferation of epidermal keratinocytes *in vitro*. In addition, the controlled release and the therapeutic efficacy of GMS@EVs were also observed in the treatment of diabetic wounds. Transcriptomic analysis illustrated that the wound healing-related genes were significantly upregulated and the pro-inflammatory genes as well as the wound healing-impaired genes were downregulated. Collectively, this study introduces a ‘ready-to-use’ platform for EV isolation and controlled release, providing a promising strategy for diabetic wound repair.

## Graphical abstract

**Graphical Abstract:**
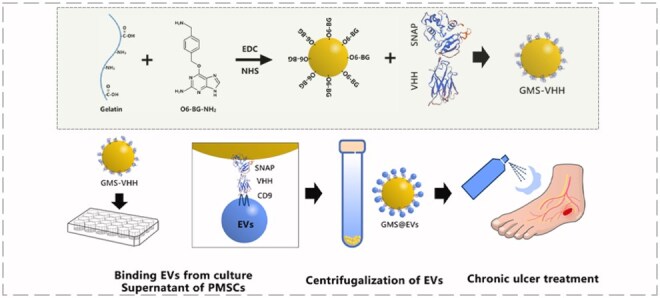

## Introduction

Diabetes is a prevalent chronic disease, with studies indicating a substantial global increase in both the number of cases and the age-standardized rate (ASR) between 1990 and 2017. In 2017, the global prevalence reached 8.8%, and this figure is projected to rise to 9.9% by 2045, underscoring its serious impact as a public health issue [1, 2]. Among this population, impaired wound healing represents one of the most common and serious complications with pathogenesis involving vasculopathy, persistent inflammation and cellular dysfunction, which collectively impede the normal healing process [3–5]. Conventional managements, such as debridement, antibiotics and regular dressing changes, are primarily aimed at controlling infection and do little to actively promote wound closure. Diabetic patients have a 19–34% lifetime risk of foot ulceration, with each ulcer carrying ∼20% risk of lower-limb amputation [4–6]. The high prevalence and substantial economic burden associated with diabetic wounds underscore an urgent need for novel and more effective therapeutic strategies.

In recent years, advances in regenerative medicine have positioned stem cell-based therapies and their derivatives as a promising therapeutic strategy for a range of degenerative diseases and injuries. Among these, mesenchymal stem cell (MSC)-deriving extracellular vesicles (EVs) have emerged as a leading candidate for wound repair and regeneration, with applications spanning renal, hepatic, cardiovascular, neurological and dermatological fields [7–9]. First identified in the early 1980s, EVs were initially regarded as by-products of receptor recycling and exocytosis. They are now recognized as sophisticated messengers that transport lipids, proteins and nucleic acids, and play important roles in immune regulation, development and tumor metastasis [8–10]. Moreover, unlike whole cells, EVs possess a streamlined membrane protein profile and lack many immunostimulatory factors, conferring inherent immune-evasive properties that make them highly attractive for regenerative medicine [11, 12]. In the context of diabetic foot ulcer (DFU) research, multiple studies have confirmed that MSC-EVs significantly enhance diabetic wound healing through anti-inflammatory, pro-angiogenic and pro-regenerative effects on epidermal and dermal cells [3, 8]. For instance, our previous work demonstrated that EVs derived from M2 macrophages promote macrophage polarization from the M1 to the M2 phenotype and stimulate fibroblast migration, thereby facilitating diabetic wound repair [13]. Additionally, hypoxia-conditioned MSC-derived EVs have been shown to deliver miR-17-5p, which suppresses excessive neutrophil extracellular trap (NET) formation and further promotes healing in diabetic wounds [14].

However, the tedious procedure for EV isolation remains a critical technical bottleneck, limiting their broader application. Conventional methods such as ultracentrifugation, precipitation and density gradient centrifugation are still widely employed, and ultracentrifugation remains the most common technique. Yet, this method is labor-intensive and time-consuming, and its dependence on mass-based separation often compromises the purity of EVs, and potentially damaging their structure and impairing bioactivity [15–17]. In recent years, emerging approaches including microfluidics, immunoaffinity capture and ultrafiltration have attracted interest due to their enhanced efficiency and specificity. These methods better preserve the structural integrity and biological activity of EVs by integrating nanotechnology and bioengineering strategies [17, 18]. However, challenges such as operational complexity, high equipment demands and elevated costs continue to restrict their routine and clinical translation [18, 19]. CD9, a key tetraspanin family protein, is stably and highly expressed on the EVs membrane, rendering it an ideal target [20, 21]. Building on advances in protein-oriented immobilization technologies [22, 23], we propose a conjugation-based isolation strategy designed to achieve high purity, preserve bioactivity and maintain cost-effectiveness for downstream research and clinical applications.

Beyond isolation, EVs-based therapies face another major challenge: poor retention and persistence at the target site. Studies indicate that simply injecting EVs locally results in suboptimal pharmacokinetics, as they are rapidly cleared through two primary mechanisms. First, they are readily washed away by circulating wound fluids (e.g. blood and lymph). Second, they are recognized and phagocytosed by innate immune cells, particularly macrophages. Together, these processes drastically shorten the effective residence time of EVs in the lesion, preventing them from reaching and sustaining the therapeutic threshold—thereby significantly compromising treatment efficacy [9, 24].

Gelatin, a natural collagen-derived biomaterial, serves as an ideal substrate for drug delivery systems due to its excellent biocompatibility, tunable biodegradation and facile functionalization [25, 26]. Its utility is further enhanced when fabricated into gelatin microspheres (GMS), which are widely recognized as excellent drug carriers [26]. GMS leverages a three-dimensional porous architecture to efficiently load a range of therapeutics—including drugs, proteins and EVs—through physical adsorption and confinement. The subsequent release of these agents, governed by gelatin degradation, enables sustained local delivery, thereby maintaining therapeutic levels over time [26, 27].

Addressing these challenges, we developed a gelatin microsphere delivery system (GMS-VHH) for the integrated isolation and sustained release of EVs. The GMS-VHH platform was assembled from three key components: GMS, the linker molecule O6-BG-NH_2_ [28, 29] and a SNAP-VHH fusion protein. The SNAP-tag domain covalently immobilizes the heavy-chain-only antibodies (VHH) onto the microspheres via its specific reaction with O6-BG [30, 31], while the VHH nanobody domain selectively targets CD9 on EVs with high affinity [32]. This configuration enabled efficient, one-step capture of EVs from MSC supernatants, forming GMS@EVs complexes that were easily collected by low-speed centrifugation. Functionally, GMS@EVs provided sustained release kinetics, promoted *in vitro* cell proliferation and markedly enhanced wound healing in a diabetic mouse model. Thus, our system combines the dual advantages of efficient EVs isolation and local sustained release; its modular design also provides a scalable technical platform for the controlled loading and delivery of other therapeutic biomolecules, underscoring its broad applicability across various disease contexts.

## Experimental section

### Preparation and characterization of GMS-VHH

All materials and solvents were purchased from commercial sources (Aladdin, Shanghai, China) and used as received.

#### Preparation of GMS

Type A gelatin with a gel strength of approximately 250 g Bloom was dissolved in deionized water to prepare a 10% (w/v) gelatin solution. Followed by added 500 μM O6-[4-(aminomethyl)benzyl]guanine (O6-ABG), 5 mg/mL 1-ethyl-3-(3-dimethylaminopropyl)carbodiimide (EDC) and 5 mg/mL N-hydroxysuccinimide (NHS), respectively. Then, 10 mL of the gelatin solution was emulsified in 40 mL of olive oil using a motor-driven stirrer for 15 min at room temperature, during which the gelatin cross-linked to form microspheres. The resulting opalescent mixture was transferred into 200 mL of a washing solution consisting of 50 mM Tris-Base and 2% sodium dodecyl sulfate (SDS), followed by shaking and washing at 37°C for 1 h. Subsequently, the aqueous mixture was centrifuged at 1000*g* to collect GMS. The harvested GMS were washed three times with 20% isopropanol to remove residual oil and stored in 70% ethanol at 4°C. Finally, the prepared GMS were classified into three size groups using filtration screens: <50 μm, 50–100 μm and 100–200 μm.

#### SNAP-VHHs expression and purification

VHH sequences were synthesized and cloned into a customized pSNAP-6His vector harboring a T7 promoter, an N-terminal SNAP tag and a C-terminal thrombin cleavage site followed by a 6× His tag. Recombinant antibodies were expressed in *Escherichia coli* BL21 (DE3) cells (TIANGEN Biotech, Beijing, China) transformed with a plasmid for the expression of sulfhydryl oxidase and DsbC. Briefly, 2 mL of an overnight pre-culture was used to inoculate 1000 mL of LB broth containing appropriate antibiotics. The cells were grown at 37°C until the OD600 reached 0.4. Sulfhydryl oxidase and DsbC expression were induced by adding 0.5%(w/v) arabinose for 1 h and the temperature was lowered to 27°C. VHH expression was induced with 0.05 mM IPTG, and the culture was further incubated overnight at 20°C. The cells were then harvested by centrifugation and stored at −80°C.

The cell pellet was resuspended in 50 mL of lysis buffer (20 mM PBS, pH 7.3, 500 mM NaCl, 2.5 mM MgCl_2_, 0.5 mg/mL lysozyme, 2 U/mL DNase I and 1 mM PMSF) and incubated for 30 min at room temperature. The suspension was subsequently sonicated and centrifuged at 18 000*g* for 20 min at 4°C. The supernatant was filtered and loaded onto a 5 mL HisTrap HP^®^ column connected to an ÄKTA pure chromatography system (Cytiva, Buckinghamshire, UK). Analytical and preparative size-exclusion chromatography were performed using Superdex™ 75 Increase 5/150 GL and Superdex™ 75 10/300 GL columns (Cytiva), respectively. Antibody concentration was determined by the BCA colorimetric assay. Purified SNAP-VHHs were analysed by SDS-PAGE on 4–20% gradient gels under denaturing conditions and visualized with Coomassie Brilliant Blue R-250 staining using a Gel Doc™ XR+ system (Bio-Rad).

#### Preparation and characterization of GMS-VHH

To prepare GMS-VHH conjugates, GMS were incubated with SNAP-VHHs at 37°C for 1 h under constant shaking. The products were then collected by centrifugation at 1000*g*. The recovery efficiency was determined by the weight ratio of the pelleted microspheres to the initial input. Furthermore, the morphology of the GMS-VHH conjugates was characterized using a laser scanning confocal microscope (Olympus FV1200-IX83, Japan).

### Preparation and characterization of GMS@EVs

To prepare GMS@EVs, Placental Mesenchymal Stem Cell (PMSC) culture supernatant and GMS-VHH microspheres were co-incubated at 37°C for 1 h under constant shaking. The products were then collected by centrifugation at 1000*g*. Subsequently, images of the obtained GMS@EVs were acquired using laser scanning confocal microscopy (FV1200-IX83, Olympus, Japan) and scanning electron microscopy (S-4300, Hitachi, Japan), respectively.

### Isolation and characterization of EVs

PMSCs were cultured in a MSC-specific medium (Youkang, NC0103) at 37°C in a humidified atmosphere of 5% CO_2_. The medium was refreshed every 2–3 days, and cells were passaged upon reaching approximately 80% confluence. For the ultracentrifuge group, EVs were isolated by ultracentrifugation using an ultracentrifuge (Optima XPN-100, Beckman Coulter, USA) via differential ultracentrifugation (2000*g* 10 min; 10 000*g* 30 min; 100 000*g* 75 min). The resulting EVs pellet was resuspended in 1× PBS, filtered through a 0.22 μm membrane and stored at −80°C. EVs used in other experiments in this study were isolated by the GMS-VHH method and then eluted from microspheres using glycine-HCl buffer (10 mM glycine, HCl adjusted to pH 2.5–3). The morphology of EVs isolated by ultracentrifugation and the GMS-VHH method was observed using transmission electron microscopy (HT7800, Hitachi, Japan). Particle size and concentration were measured by nanoparticle tracking analysis (ZetaView, Particle Metrix, Germany).

### EVs identification (Western blot)

EVs were identified by Western blot following standard procedures. Proteins were probed with the following primary antibodies: Calnexin (ABclonal, A15631; 1:1000), CD63 (Proteintech, 25682-1-AP; 1:1000), CD9 (Proteintech, 20597-1-AP; 1:1000) and TSG101 (Proteintech, 28283-1-AP; 1:1000), followed by incubation with HRP-conjugated secondary antibodies (Zsbio, ZB-2305 or ZB-2301; 1:10 000). Signals were developed with an ECL reagent (Biosharp, BL520A) and captured using a chemiluminescence imager (Alliance MINI HD9, UVITEC, UK).

### Cell viability assay

HaCaT cells were seeded in the lower chamber of a 24-well Transwell plate at a density of 2 × 10^4^ cells per well. The upper chamber was then supplemented with complete medium, 20 µL of GMS@EVs (prepared from 20 mL of PMSC supernatant), an equivalent dose of free EVs (eluted from 20 µL of the GMS@EVs formulation; at a particle concentration of 3.5 × 10^10^/mL) or GMS alone. After 48 h of co-culture, 20 μL of CCK-8 reagent (Beyotime, C0038) was added to each well containing 200 μL of culture medium. Following incubation at 37°C for 1 h, the absorbance at 450 nm was measured using a microplate reader (SPARK 10M, TECAN, Switzerland).

### Animal experiments

#### Ethics statement

This study utilized mice obtained from Beijing Viewsolid Biotechnology Co., Ltd. All animal protocols followed the National Research Council’s Guide for the Care and Use of Laboratory Animals, and were approved by the Animal Welfare and Ethics Committee of Beijing Viewsolid Biotechnology Co., Ltd (Ethics committee approval code: VS25123101). We made every effort to minimize suffering and reduce the number of animals required.

#### *In vivo* imaging

Luciferase-labeled EVs and GMS@EVs were subcutaneously injected into the left and right flanks of 7-week-old nude mice, respectively. Subsequently, a chemiluminescence imager (Alliance MINI HD9, UVITEC, UK) was used for bioluminescence imaging immediately after injection, and at 0.5, 12 and 48 h post-injection.

#### Wound healing assessment in diabetic mice

The dorsal skin of diabetic mice was shaved, and a full-thickness circular wound with a diameter of 1 cm was created. The mice were randomly assigned to four groups, and each wound was treated topically with 100 µL of the following: PBS (control), GMS@EVs (prepared from 100 mL of PMSC supernatant), an equivalent dose of free EVs (eluted from 100 µL of the GMS@EVs formulation; at a particle concentration of 3.5 × 10^10^/mL) or GMS alone. Wound areas were photographed on days 0, 3, 7, 14 and 21 and quantified using ImageJ. For hematoxylin and eosin (H&E) staining, wound tissue samples were harvested on days 7, 14 and 21. Additionally, major organs (heart, liver, spleen, lung and kidney) were harvested on day 14. All tissue samples underwent fixation, dehydration, embedding, sectioning and staining according to standard protocols. Stained sections were scanned using a Pannoramic MIDI scanner (3DHISTECH, Hungary). The re-epithelialization rate, thickness of epidermis, granulation tissue thickness and hair follicle regeneration were quantified using ImageJ.

#### Transcriptome sequencing and bioinformatics analysis

Diabetic mice were treated as described previously. For transcriptomic analysis, wound center tissues from each group were collected on day 14, rinsed with DEPC-treated water and snap-frozen in liquid nitrogen. RNA extraction, library preparation (TIANSeq, Illumina platform), paired-end 150 bp sequencing and subsequent bioinformatic analyses were performed by LC SCIENCES (Hangzhou, China) following the manufacturer’s standard protocol. Differentially expressed genes (DEGs) were identified using DESeq2 with the thresholds of FDR <0.05 and |log_2_ fold change| ≥ 2. Gene ontology (GO) and KEGG pathway enrichment analyses were conducted using default parameters to calculate enrichment scores and *P* values.

#### Hemolysis assay

Whole blood was collected from C57BL/6J mice in EDTA-precoated tubes. Red blood cells (RBCs) were isolated by centrifugation at 500*g* for 10 min and washed five times with PBS lacking calcium and magnesium. The purified RBCs were resuspended to a density of 2 × 10^8^ cells/mL and mixed at a 1:1 (v/v) ratio with 100 μL of GMS, EVs or GMS@EVs. Deionized water and PBS were used as the positive and negative controls, respectively. All mixtures were incubated at 37 °C for 3 h with gentle shaking. After incubation, the samples were centrifuged again. The supernatant was transferred to a 96-well plate, and absorbance was measured at 540 and 655 nm using a microplate reader (SPARK 10M, TECAN, Switzerland). The percentage of hemolysis was calculated according to the following formula:


$$\mathrm{Hemolysis}(\%)=[(A_{s}-A_{n}c)/(A_{p}c-A_{n}c)]\times 100\%$$

where *A*ₛ, *A*ₙc and *A*ₚc represent the difference in absorbance (540–655 nm) for the test sample, negative control and positive control, respectively.

### Statistical analysis

Data are presented as the mean ± standard deviation (SD) from at least three independent experiments. For comparisons among multiple groups, one-way analysis of variance (ANOVA) was employed, followed by Bonferroni’s *post hoc* test. Statistical significance was defined as *P < 0.05, **P < 0.01, ***P < 0.001 and ****P < 0.0001.

## Results

### Preparation and characterization of GMS-VHH

**Figure 1:**
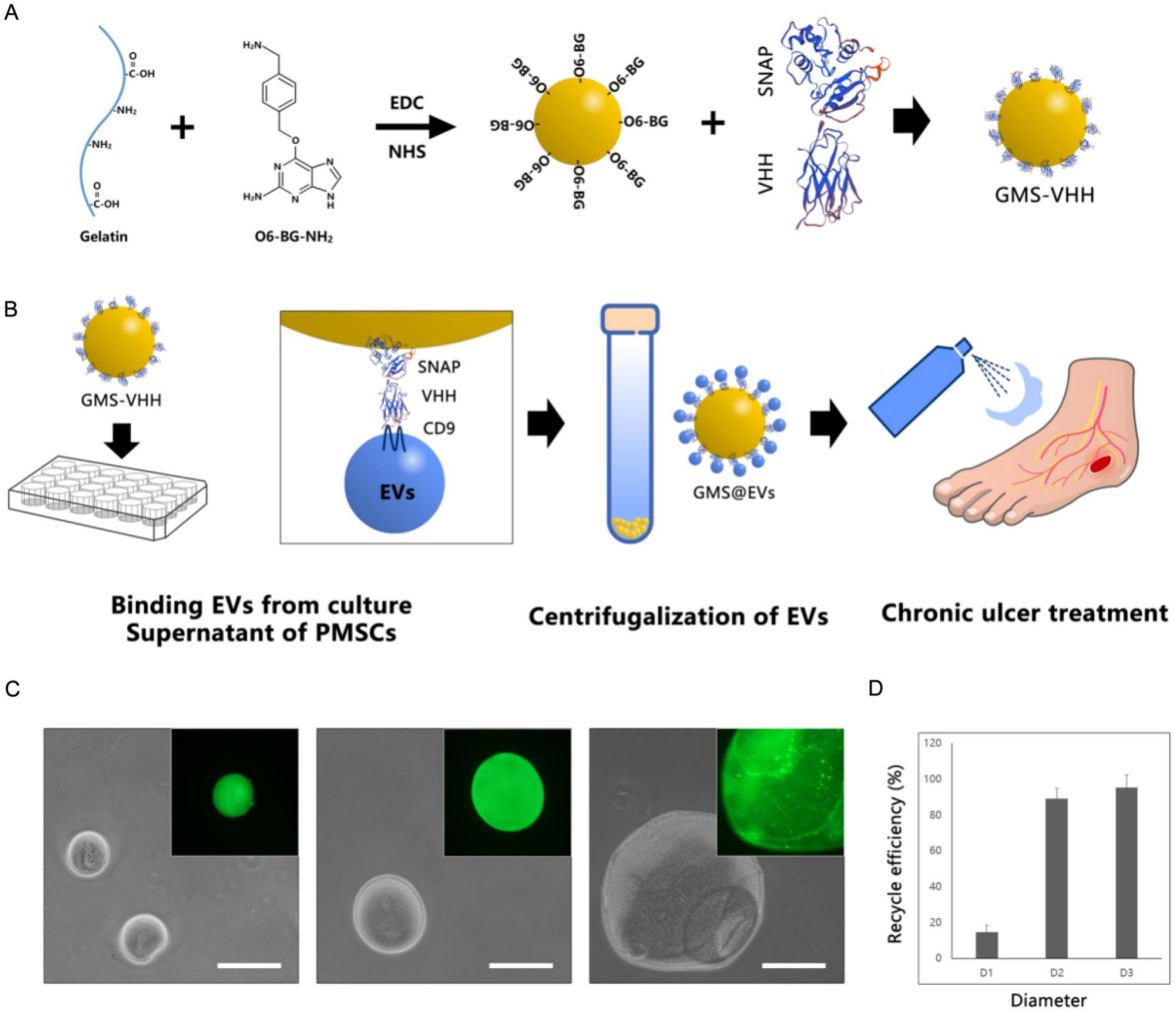
Preparation and characterization of GMS-VHH. (**A**) Schematic illustration of GMS-VHH fabrication: a blend of gelatin and O6-BG-NH2 is crosslinked into microspheres via EDC/NHS chemistry, followed by co-incubation with SNAP-tagged antibodies to enable covalent conjugation of antibodies onto the microsphere surface. (**B**) Schematic workflow of GMS-VHH application: the microspheres are incubated with cell culture supernatant to capture CD9-positive EVs. EVs-bound gelatin microspheres (GMS@EVs) are then collected by simple centrifugation and sprayed onto wounds for therapeutic purposes. (**C**) Bright-field images of GMS-VHH with varying sizes, with fluorescence images (inset) depicting effective binding of SNAP-tag fluorescent antibodies, scale bar = 100 μm. (**D**) Centrifugal recovery efficiency of GMS-VHH across different sizes. The recovery rate approaches 100% when the microsphere diameter exceeds 100 μm.

As shown in Figure 1A, we employed an EDC/NHS-mediated crosslinking strategy to conjugate gelatin with the SNAP-tag substrate O6-BG-NH_2_, forming GMS. These microspheres were then incubated with SNAP-tagged CD9 nanobodies (SNAP-VHH), which covalently immobilized the nanobodies onto the microsphere surface via the specific reaction between the SNAP-tag and O6-BG-NH_2_, yielding GMS-VHH (Figure 1A). The VHH is a nanobody that can recognize CD9 on the surface of EVs with high affinity. When fused to a SNAP-tag, it enables site-specific immobilization without loss of specificity [32, 33]. CD9, as a member of the tetraspanin family, is widely expressed on the EVs membrane and participates in intercellular communication and signal transduction [20, 21]. Based on this design, GMS-VHH effectively captures EVs from culture supernatants with high specificity and stability. The EVs-bound microspheres (GMS@EVs) can then be isolated by low-speed centrifugation and directly applied for therapeutic purposes (Figure 1B). Using this approach, we successfully fabricated GMS-VHH of various sizes. These microspheres exhibited uniform morphology and effectively bound SNAP-tagged fluorescent antibodies as confirmed by confocal fluorescence imaging, confirming successful surface functionalization (Figure 1C). Evaluation of the centrifugation recovery efficiency revealed that microspheres exceeding 100 μm in size could be recovered nearly completely (Figure 1D). Therefore, this size fraction was chosen for all subsequent studies.

### Characterization of GMS@EVs

**Figure 2:**
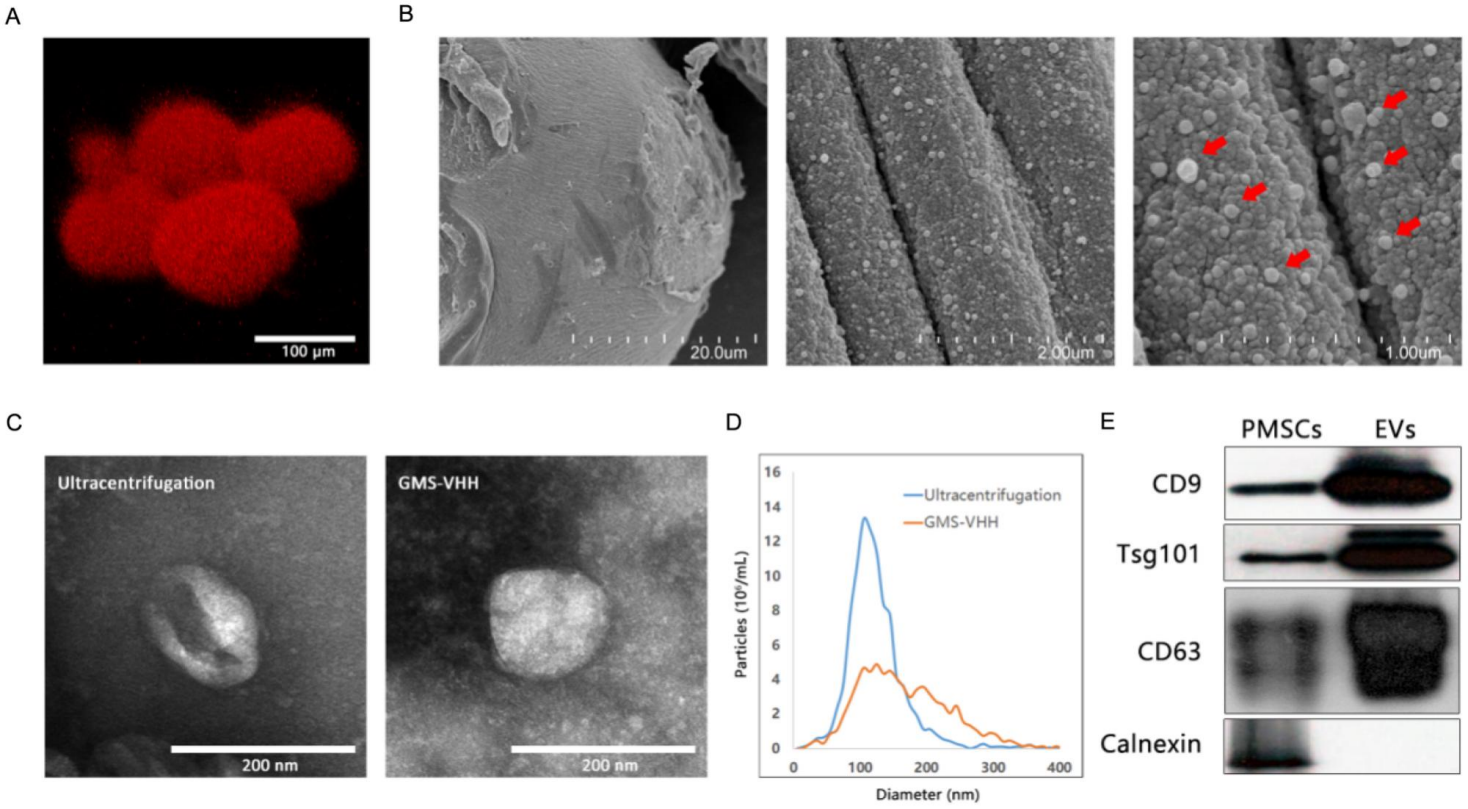
Characterization of GMS@EVs. (**A**) Confocal laser scanning microscopy (CLSM) image of GMS@EVs with robustly captured Dil-labeled EVs. (**B**) Scanning electron microscopy (SEM) image revealing numerous EVs (arrows) embedded within the surface folds of a microsphere. (**C**) Representative transmission electron microscopy (TEM) images comparing the morphology of EVs isolated by traditional ultracentrifugation (left) versus the GMS-VHH method (right). (**D**) Nanoparticle tracking analysis (NTA) comparing the size distribution of EVs isolated by traditional ultracentrifugation and the GMS-VHH method. (**E**) Western blot analysis of EVs-specific markers in proteins derived from PMSC and EVs isolated using GMS-VHH.

To evaluate the EVs-isolation capability of GMS-VHH, we labeled PMSC-derived EVs with the Dil fluorescent dye. Confocal microscopy revealed that the Dil-labeled EVs exhibited red fluorescence and were uniformly distributed on the surface of the GMS (Figure 2A), indicating successful capture and stable binding of EVs. Further detailed observation via scanning electron microscopy demonstrated that a large number of EVs, approximately 200 nm in diameter, were embedded within the folded structures on the microsphere surface (Figure 2B). This distribution pattern not only reflects the efficient adsorption capacity of GMS toward EVs but also implies potential application value: the porous surface structure may facilitate the adsorption and sustained release of EVs, thereby prolonging their retention and action at the local wound site [34].

We next compared the morphology and size distribution of EVs isolated by traditional ultracentrifugation and the GMS-VHH method. Ultracentrifugation, a commonly used method for EVs isolation, is prone to cause damage to EVs membrane structure during high-speed centrifugation, which may compromise their bioactivity [15–17]. Transmission electron microscopy results (Figure 2C) showed that, in contrast to the frequently observed ‘cup-shaped’ EVs in the ultracentrifugation group, EVs captured by GMS-VHH appeared more rounded and structurally intact, suggesting better preservation of EVs structural integrity with this method. Nanoparticle tracking analysis (Figure 2D) indicated that although the overall size of EVs isolated by GMS-VHH was slightly larger than those from the ultracentrifugation group, the majority still fell within the typical EVs size range of 100–200 nm. Furthermore, Western blot analysis (Figure 2E) confirmed that EVs captured by GMS-VHH highly expressed EVs marker proteins CD9, TSG101 and CD63, while negative markers such as Calnexin were not detected, supporting the high purity and typical characteristics of the isolated EVs.

Collectively, the GMS-VHH isolation method outperforms traditional ultracentrifugation in maintaining EVs structural integrity and enhancing isolation efficiency. This approach offers a new technical strategy for EVs-related research and provides a solid foundation for subsequent experimental exploration and clinical application.

### Bioactivity and sustained-release performance of GMS@EVs

**Figure 3:**
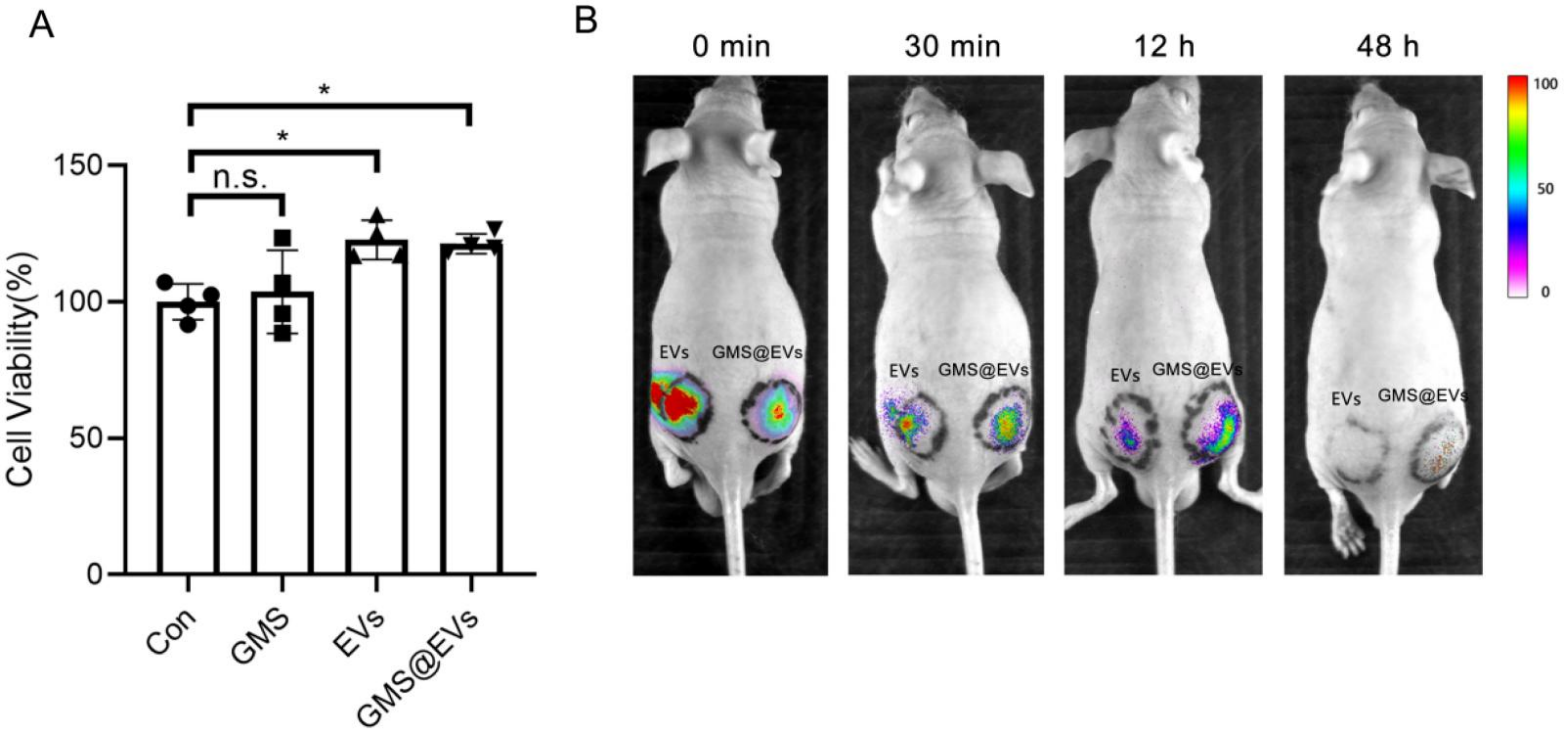
Bioactivity and sustained-release performance of GMS@EVs. (**A**) Viability of HaCaT cells treated with GMS@EVs, and corresponding concentrations of GMS and EVs alone, as determined by CCK-8 assay. *P < 0.05. (**B**) *In vivo* fluorescence imaging of nude mice at different time points. Free luciferase-labeled EVs were injected into the left buttock, while GMS@EVs were injected into the right buttock.

Biocompatibility is a critical benchmark for biomaterials used in biomedical applications. Meanwhile, EVs-based therapies still face challenges of short *in vivo* retention time [9, 24]. Therefore, we further evaluated the biocompatibility and *in vivo* sustained-release profile of GMS@EVs. As illustrated in Figure 3A, CCK-8 assays revealed that the GMS group exhibited good biocompatibility compared with the control group, while both the EVs and GMS@EVs groups significantly enhanced cell proliferation. In the *in vivo* release study, luciferase-labeled EVs were administered subcutaneously to nude mice either in free form or encapsulated in GMS@EVs, and their distribution was monitored via *in vivo* fluorescence imaging (Figure 3B). The results demonstrated that free EVs dispersed rapidly within 30 min after injection, with a marked reduction in signal after 12 h and near complete disappearance by 48 h. In contrast, the GMS@EVs group maintained a strong fluorescence signal over an extended period, with a clearly detectable signal after 48 h. This result indicates that GMS@EVs can effectively retard the release of EVs and prolong their local duration of action. Our findings demonstrate that GMS@EVs exhibit favorable biocompatibility and improve *in vivo* retention, providing a robust strategy for the sustained local delivery of EVs.

### GMS@EVs promote wound healing in diabetic mice

**Figure 4:**
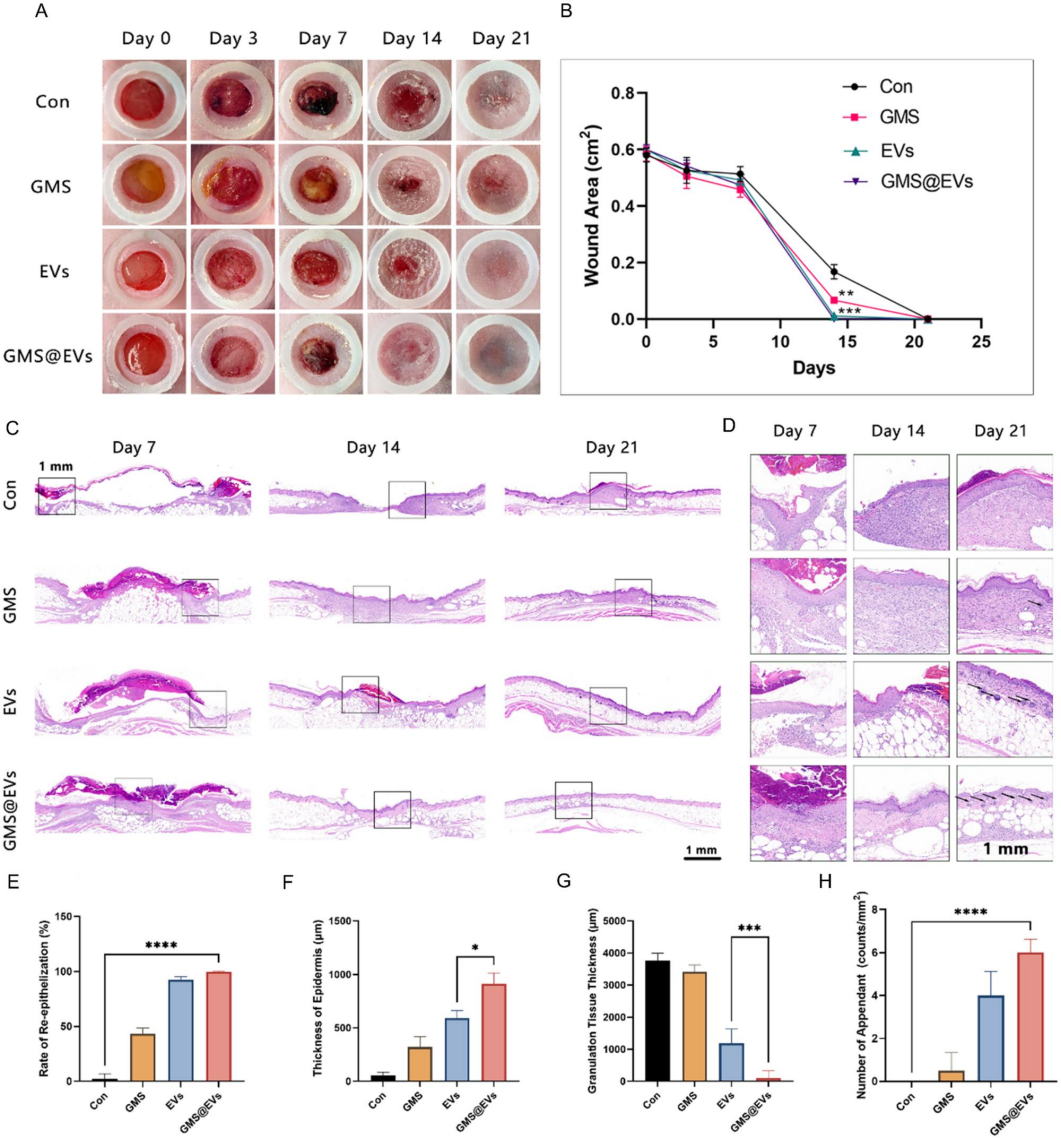
GMS@EVs promote wound healing in diabetic mice. (**A**) Representative photographic images and (**B**) quantitative analysis of wounds in diabetic mice treated with PBS, GMS, EVs and GMS@EVs on days 0, 3, 7, 14 and 21. (**C, D**) Representative hematoxylin and eosin (H&E)-stained sections of wounds from different treatment groups on days 7, 14 and 21. Quantitative analysis of (**E**) re-epithelialization rate, (**F**) thickness of the neo-epithelium at wound center, (**G**) granulation tissue thickness at day 14 and (**H**) number of regenerated hair follicles at day 21. *P < 0.05, **P < 0.01, ***P < 0.001, ****P < 0.0001.

We further evaluated the therapeutic efficacy of GMS@EVs in a diabetic mouse wound model. Full-thickness skin defects were created on the backs of db/db diabetic mice and treated with PBS, GMS, EVs or GMS@EVs respectively. As illustrated in Figure 4A and B, wound healing was markedly enhanced in the GMS@EVs group compared to the other groups, followed by the EVs and GMS-alone groups. In the early stages of healing, the GMS@EVs group already exhibited a clear repair advantage, characterized by cleaner wound margins, active granulation tissue formation and significantly accelerated wound closure. By day 14, wounds in the GMS@EVs group had nearly completely healed, whereas visible signs of healing were not observed until day 21 in the control group. Notably, GMS promoted wound healing to some extent, which may be attributed to the good biocompatibility of gelatin and its inherent ability to support tissue repair.

**Figure 5:**
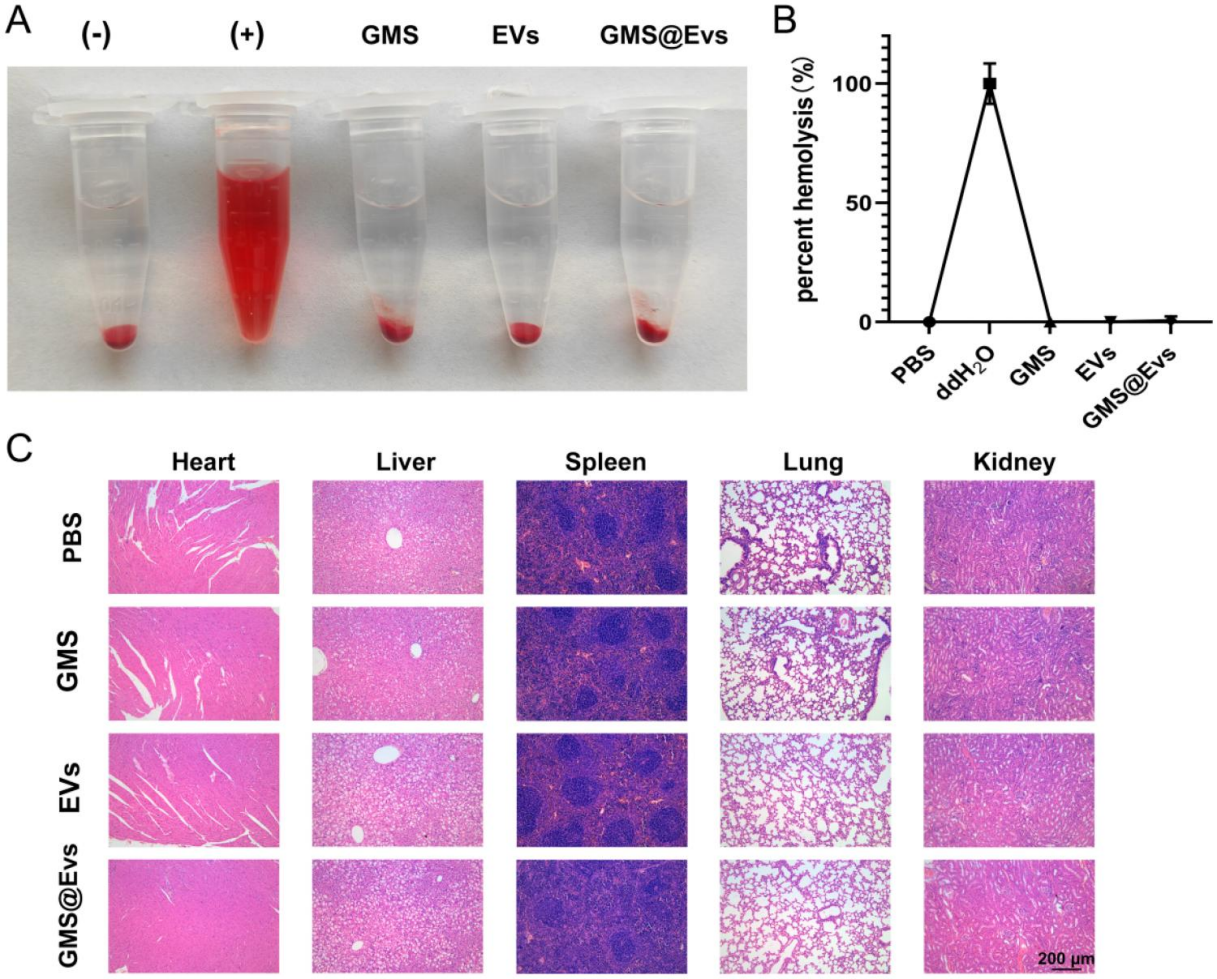
Biocompatibility and safety assessment of GMS, EVs and GMS@EVs. (**A**) Representative images of red blood cells (RBCs) after 3 h of incubation with GMS, EVs and GMS@EVs. Hemoglobin release into the supernatant indicates RBC membrane damage and RBC lysis. (+) and (−) represent positive (water) and negative (PBS) controls, respectively. (**B**) Quantitative hemolysis rate analysis (*n* = 5). (**C**) Histological evaluation of major organs harvested from diabetic mice treated with PBS, GMS, EVs and GMS@EVs, scale bar = 200 μm.

Subsequent histological evaluation (H&E staining) was performed to assess the quality of the healed tissue. In the GMS@EVs-treated wounds, newly formed and well-organized epidermis with regenerated skin appendages and complete re-epithelialization were observed (Figure 4C and D), while the control group exhibited delayed re-epithelialization. Quantitative analysis (Figure 4E–H) revealed that the GMS@EVs group was significantly superior to the control and other experimental groups in terms of re-epithelialization rate (Figure 4E), epidermis thickness (Figure 4F) and granulation tissue thickness (Figure 4G). Additionally, the GMS@EVs group showed enhanced regeneration of hair follicles (Figure 4H), indicating that GMS@EVs treatment not only accelerated wound closure but also promoted structurally and functionally superior tissue regeneration. These findings demonstrate that GMS@EVs significantly enhance both the rate and quality of diabetic wound healing. Furthermore, the hemolysis assay demonstrated that GMS-VHH did not induce significant hemolysis, and no obvious histopathological changes were observed in the major organs of diabetic mice treated with GMS-VHH, which supports the safety of its *in vivo* application (Figure 5).

### Transcriptomic analysis of GMS@EVs-mediated wound healing

**Figure 6:**
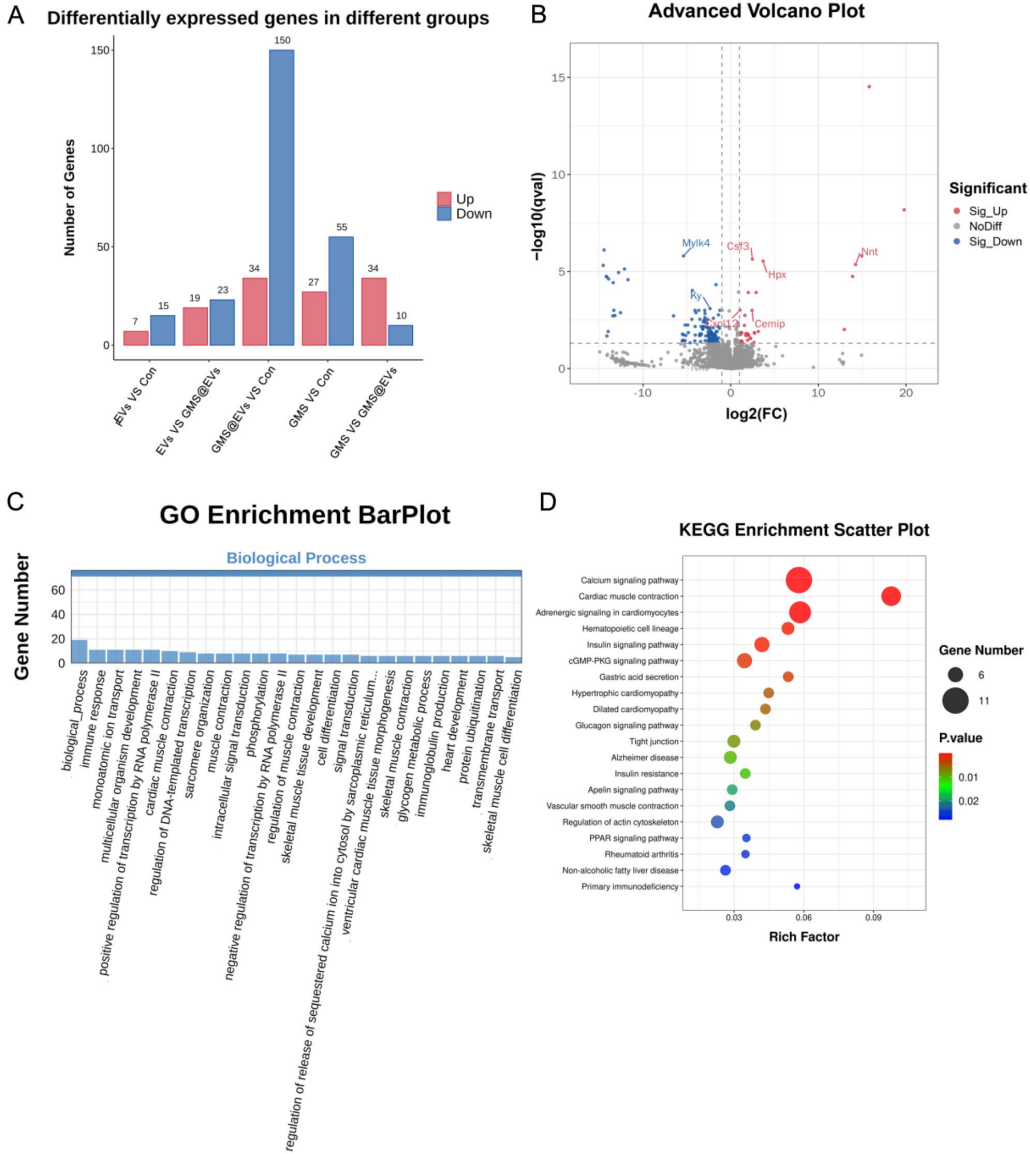
Transcriptomic analysis of GMS@EVs-mediated wound healing. (**A**) Overview of differential gene expression across groups. (**B**) Volcano plot displaying differentially expressed genes (DEGs) between the GMS@EVs and PBS groups. (**C**) Gene ontology (GO) biological process terms enriched in DEGs from the GMS@EVs vs. PBS comparison. (**D**) Significantly enriched KEGG pathways identified from DEGs between the GMS@EVs and PBS groups.

To gain deeper insight into the molecular mechanisms by which GMS@EVs promote wound healing, we performed RNA sequencing (RNA-seq) on wound tissues harvested from each group on day 14. As illustrated in Figure 6A, transcriptomic profiling revealed widespread differential gene expression across the groups, with the GMS@EVs group showing the highest number of DEGs relative to the control. Among 15 654 detected genes, a total of 184 genes were identified as differentially expressed (34 upregulated and 150 downregulated). Notably, several genes associated with pro-regenerative processes—such as Cxcl12, Nnt, Cemip and Acaa1c—as well as genes involved in oxidative stress resistance and infection control—including Hpx and Csf3—were significantly upregulated. In contrast, genes with potential pro-inflammatory or repair-impairing roles, such as Mylk4 and Ky, were downregulated (Figure 6B).

Gene ontology (GO) enrichment analysis further demonstrated that the most significantly enriched biological processes were related to inflammation, metabolism, cell proliferation and differentiation, and tissue reconstruction (Figure 6C). Kyoto Encyclopedia of Genes and Genomes (KEGG) pathway analysis highlighted the involvement of several key pathways, including the cGMP-PKG, Apelin and HIF-1 signaling pathways—all known to be critical for improving hypoxia, vascular function and inflammatory responses—in addition to the regulation of actin cytoskeleton and arginine and proline metabolism pathways, which are implicated in cell migration, repair and protein synthesis (Figure 6D) [35–37].

Collectively, these transcriptomic findings confirm at the molecular level that GMS@EVs effectively facilitate wound healing by synergistically modulating multiple key biological processes and signaling pathways. The observed gene expression changes not only reflect the multi-faceted regulatory role of GMS@EVs in wound repair but also offer potential therapeutic targets for future investigation.

## Discussion

The healing of diabetic wounds is a clinically complex and challenging process, for which current therapeutic strategies often fail to ensure timely and complete closure [38]. This highlights an urgent need for more effective interventions. In this context, EVs derived from stem cells, known for their remarkable regenerative and reparative properties, have emerged as a highly promising cell-free therapeutic tool in regenerative medicine [39]. Multiple studies have demonstrated their ability to significantly accelerate diabetic wound healing [3, 8, 10]. To overcome the challenges associated with the complex, time-consuming isolation and rapid *in vivo* clearance of EVs, this study developed a GMS-VHH system that integrates specific EV capture, one-step purification and localized sustained release into a single platform. By addressing these key bottlenecks in EV processing and delivery, the system not only improves the practical applicability of EV-based therapies but also offers a novel strategy to enhance diabetic wound healing outcomes.

Gelation was selected as the matrix owing to its natural origin, excellent biocompatibility, biodegradability and the presence of abundant amino and carboxyl groups along its molecular chain, which facilitate chemical modification [26, 40]. Using an EDC/NHS crosslinking reaction, we covalently grafted the SNAP-tag substrate O6-BG-NH_2_ onto the surface of GMS. This design leverages the efficient, mild and specific covalent reaction between O6-BG-NH_2_ and SNAP-VHH fusion proteins, enabling stable anchoring of the targeting unit onto the microspheres for precise capture of EVs [31]. In contrast to traditional ultracentrifugation—which is labor-intensive and may compromise EVs structural integrity—the GMS-VHH method allows the preparation of ‘ready-to-use’ therapeutic microspheres through simple incubation and low-speed centrifugation, significantly streamlining the process. Experimental results confirmed that EVs isolated using the GMS-VHH method exhibit superior structural integrity compared with those obtained by ultracentrifugation. More importantly, as a biodegradable material, the GMS@EVs enable localized, long-term and sustained release of the loaded EVs as the matrix gradually degrades. *In vivo* imaging data clearly demonstrated that GMS@EVs form a long-acting ‘drug depot’ at the injection site, effectively preventing rapid EVs clearance and significantly prolonging their *in vivo* retention, thereby establishing the foundation for sustained therapeutic efficacy [41].

Leveraging the inherent tissue-supporting properties of gelatin and its capacity for controlled EVs release, the GMS@EVs system demonstrated significant therapeutic benefits for wound healing [42, 43]. In a diabetic mouse wound model, the GMS@EVs group exhibited the most pronounced healing response. Histological analysis further revealed that the treatment not only markedly accelerated re-epithelialization—yielding a neo-epithelium with a thickness comparable to normal skin—but also promoted the regeneration of skin appendages, including hair follicles. These findings indicate a shift in the healing mechanism from rudimentary ‘scar repair’ to a more advanced ‘functional regeneration’. Furthermore, at the pro-healing dosage, GMS@EVs enhanced the proliferation of HaCaT cells *in vitro*, and no systemic toxicity was observed following local application in mice. Collectively, this study highlights the promising potential of GMS@EVs as a highly effective and safe therapeutic dressing for diabetic wound treatment.

To further elucidate the underlying mechanisms *in vivo*, we performed transcriptomic sequencing. The analysis revealed that the superior efficacy of GMS@EVs was attributable to the coordinated regulation of a complex gene network. This network broadly encompasses multiple biological processes and signaling pathways closely associated with tissue repair. We propose that the localized and sustained ‘EVs reservoir’ formed by GMS@EVs ensures the continuous and stable release of EVs on the wound microenvironment during the critical repair period, thereby initiating a synergistic and sequential molecular cascade that ultimately drives high-quality tissue regeneration.

While this study has yielded promising results, several challenges must be addressed to advance the clinical translation of this strategy. First, it is essential to establish a scalable manufacturing process for GMS and SNAP-VHH fusion proteins that complies with good manufacturing practice (GMP) standards, along with rigorous quality control protocols to ensure batch-to-batch consistency. Second, beyond the transcriptomic insights obtained, future studies should integrate multi-omics approaches—such as proteomics and metabolomics—and employ tools such as cell-specific knockout models to more precisely delineate the core molecular targets and signaling pathways modulated by GMS@EVs [44, 45]. Such in-depth mechanistic investigation may also offer new perspectives for other areas of tissue repair and regeneration research including chronic inflammation, fibrosis and ischemic injury models.

In summary, the GMS@EVs system developed in this study provides an integrated solution to the key bottlenecks in EV isolation and delivery by providing a ready-to-use platform that greatly streamlines the entire application process. This strategy establishes a novel and promising approach for diabetic wound healing with broad potential for future EV-based therapeutics.

## Conclusion

We developed a GMS-VHH system for rapid capture, ready-to-use preparation and controlled release of EVs. The system facilitates specific EV capture through conjugation of VHH nanobodies that target the highly expressed surface marker CD9 on EVs. By leveraging the biodegradable nature of GMS, the platform enables sustained EV release. PMSC-deriving EVs isolated using this method exhibited good biocompatibility and enhanced cellular proliferation *in vitro*. In diabetic mice, GMS@EVs significantly accelerated wound healing, accompanied by improved re-epithelialization and hair follicle regeneration, thereby offering a novel therapeutic strategy for refractory diabetic wounds. Notably, this platform effectively overcomes the limitations of conventional EV isolation and application methods by providing an integrated, ready-to-use strategy, highlighting its broad potential for future EV-based therapeutics.

## References

[1] Liu J , Ren ZH, Qiang H, Wu J, Shen M, Zhang L, Lyu J. Trends in the incidence of diabetes mellitus: results from the Global Burden of Disease Study 2017 and implications for diabetes mellitus prevention. BMC Public Health 2020;20:1415. 32943028 10.1186/s12889-020-09502-x PMC7500018

[2] Standl E , Khunti K, Hansen TB, Schnell O. The global epidemics of diabetes in the 21st century: current situation and perspectives. Eur J Prev Cardiol 2019;26:7–14. 10.1177/2047487319881021 31766915

[3] Wang J , Liang YJ, Pan X. Advances in the role of stem cell-derived exosomes in diabetic foot wound healing. Diabetes Metab Syndr Obes 2025;18:2767–81. 40821749 10.2147/DMSO.S521095 PMC12350548

[4] McDermott K , Fang M, Boulton AJM, Selvin E, Hicks CW. Etiology, epidemiology, and disparities in the burden of diabetic foot ulcers. Diabetes Care 2023;46:209–21. 36548709 10.2337/dci22-0043 PMC9797649

[5] Armstrong DG , Tan TW, Boulton AJM, Bus SA. Diabetic foot ulcers: a review. JAMA 2023;330:62–75. 37395769 10.1001/jama.2023.10578 PMC10723802

[6] The global, regional, and national burden of cancer, 1990–2023, with forecasts to 2050: a systematic analysis for the Global Burden of Disease Study 2023. Lancet 2025;406:1565–86. 41015051 10.1016/S0140-6736(25)01635-6 PMC12687902

[7] Tang Y , Zhou Y, Li H. Advances in mesenchymal stem cell exosomes: a review. Stem Cell Res Ther 2021;12:71. 33468232 10.1186/s13287-021-02138-7 PMC7814175

[8] Li D , Li D, Wang Z, Li J, Shahzad KA, Wang Y, Tan F. Signaling pathways activated and regulated by stem cell-derived exosome therapy. Cell Biosci 2024;14:105. 39164778 10.1186/s13578-024-01277-7 PMC11334359

[9] Tian J , Han Z, Song D, Peng Y, Xiong M, Chen Z, Duan S, Zhang L. Engineered exosome for drug delivery: recent development and clinical applications. Int J Nanomedicine 2023;18:7923–40. 38152837 10.2147/IJN.S444582 PMC10752020

[10] Mathieu M , Martin-Jaular L, Lavieu G, Théry C. Specificities of secretion and uptake of exosomes and other extracellular vesicles for cell-to-cell communication. Nat Cell Biol 2019;21:9–17. 30602770 10.1038/s41556-018-0250-9

[11] Greening DW , Xu R, Ale A, Hagemeyer CE, Chen W. Extracellular vesicles as next generation immunotherapeutics. Semin Cancer Biol 2023;90:73–100. 36773820 10.1016/j.semcancer.2023.02.002

[12] Cheng W , Xu C, Su Y, Shen Y, Yang Q, Zhao Y, Zhao Y, Liu Y. Engineered extracellular vesicles: a potential treatment for regeneration. iScience 2023;26:108282. 38026170 10.1016/j.isci.2023.108282 PMC10651684

[13] Meng H , Su J, Shen Q, Hu W, Li P, Guo K, Liu X, Ma K, Zhong W, Chen S, Ma L, Hao Y, Chen J, Jiang Y, Li L, Fu X, Zhang C. A smart MMP-9-responsive hydrogel releasing M2 macrophage-derived exosomes for diabetic wound healing. Adv Healthc Mater 2025;14(10):2404966. 10.1002/adhm.202404966 39955735

[14] Chu Z , Huang Q, Ma K, Liu X, Zhang W, Cui S, Wei Q, Gao H, Hu W, Wang Z, Meng S, Tian L, Li H, Fu X, Zhang C. Novel neutrophil extracellular trap-related mechanisms in diabetic wounds inspire a promising treatment strategy with hypoxia-challenged small extracellular vesicles. Bioact Mater 2023;27:257–70. 37122894 10.1016/j.bioactmat.2023.04.007 PMC10133407

[15] Han L , Zhao Z, He C, Li J, Li X, Lu M. Removing the stumbling block of exosome applications in clinical and translational medicine: expand production and improve accuracy. Stem Cell Res Ther 2023;14:57. 37005658 10.1186/s13287-023-03288-6 PMC10068172

[16] Heidarpour M , Krockenberger M, Bennett P. Review of exosomes and their potential for veterinary medicine. Res Vet Sci 2024;168:105141. 38218063 10.1016/j.rvsc.2024.105141

[17] Pallares-Rusiñol A , Bernuz M, Moura SL, Fernández-Senac C, Rossi R, Martí M, Pividori MI. Advances in exosome analysis. Adv Clin Chem 2023;112:69–117. 36642486 10.1016/bs.acc.2022.09.002

[18] Martínez-Santillán A , González-Valdez J. Novel technologies for exosome and exosome-like nanovesicle procurement and enhancement. Biomedicines 2023;11:1487. 37239158 10.3390/biomedicines11051487 PMC10216008

[19] Gao J , Li A, Hu J, Feng L, Liu L, Shen Z. Recent developments in isolating methods for exosomes. Front Bioeng Biotechnol 2022;10:1100892. 36714629 10.3389/fbioe.2022.1100892 PMC9879965

[20] Böker KO , Lemus-Diaz N, Rinaldi Ferreira R, Schiller L, Schneider S, Gruber J. The impact of the CD9 tetraspanin on lentivirus infectivity and exosome secretion. Mol Ther 2018;26:634–47. 29221804 10.1016/j.ymthe.2017.11.008 PMC5835022

[21] Carney RP , Hazari S, Colquhoun M, Tran D, Hwang B, Mulligan MS, Bryers JD, Girda E, Leiserowitz GS, Smith ZJ, Lam KS. Multispectral optical tweezers for biochemical fingerprinting of CD9-positive exosome subpopulations. Anal Chem 2017;89:5357–63. 28345878 10.1021/acs.analchem.7b00017 PMC5551404

[22] Fisher SA , Baker AEG, Shoichet MS. Designing peptide and protein modified hydrogels: selecting the optimal conjugation strategy. J Am Chem Soc 2017;139:7416–27. 28481537 10.1021/jacs.7b00513

[23] Steen Redeker E , Ta DT, Cortens D, Billen B, Guedens W, Adriaensens P. Protein engineering for directed immobilization. Bioconjug Chem 2013;24:1761–77. 24160176 10.1021/bc4002823

[24] Parada N , Romero-Trujillo A, Georges N, Alcayaga-Miranda F. Camouflage strategies for therapeutic exosomes evasion from phagocytosis. J Adv Res 2021;31:61–74. 34194832 10.1016/j.jare.2021.01.001 PMC8240105

[25] Milano F , Masi A, Madaghiele M, Sannino A, Salvatore L, Gallo N. Current trends in gelatin-based drug delivery systems. Pharmaceutics 2023;15:1499. 37242741 10.3390/pharmaceutics15051499 PMC10221778

[26] Bello AB , Kim D, Kim D, Park H, Lee S-H. Engineering and functionalization of gelatin biomaterials: from cell culture to medical applications. Tissue Eng Part B Rev 2020;26:164–80. 31910095 10.1089/ten.TEB.2019.0256

[27] Foox M , Zilberman M. Drug delivery from gelatin-based systems. Expert Opin Drug Deliv 2015;12:1547–63. 25943722 10.1517/17425247.2015.1037272

[28] Mottram LF , Maddox E, Schwab M, Beaufils F, Peterson BR. A concise synthesis of the Pennsylvania Green fluorophore and labeling of intracellular targets with O6-benzylguanine derivatives. Org Lett 2007;9:3741–4. 17705395 10.1021/ol7015093

[29] Ohyanagi T , Shima T, Okada Y, Tsukasaki Y, Komatsuzaki A, Tsuboi S, Jin T. Compact and stable SNAP ligand-conjugated quantum dots as a fluorescent probe for single-molecule imaging of dynein motor protein. Chem Commun (Camb) 2015;51:14836–9. 26267231 10.1039/c5cc05526a

[30] Cole NB. Site-specific protein labeling with SNAP-tags. Curr Protoc Protein Sci 2013;73:30.1.1–16. 10.1002/0471140864.ps3001s73 PMC3920298 24510614

[31] Kindermann M , George N, Johnsson N, Johnsson K. Covalent and selective immobilization of fusion proteins. J Am Chem Soc 2003;125:7810–1. 12822993 10.1021/ja034145s

[32] Tillib SV. Prospective applications of single-domain antibodies in biomedicine. Mol Biol (Mosk) 2020;54:362–73. 32492000 10.31857/S0026898420030167

[33] Heukers R , Mashayekhi V, Ramirez-Escudero M, de Haard H, Verrips TC, van Bergen En Henegouwen PMP, Oliveira S. VHH-photosensitizer conjugates for targeted photodynamic therapy of met-overexpressing tumor cells. Antibodies (Basel) 2019;8:26. 31544832 10.3390/antib8020026 PMC6640711

[34] Cao S , Li L, Du Y, Gan J, Wang J, Wang T, Liu Y, Liu W, Zhou Y, Gao X, Li H, Liu T. Porous gelatin microspheres for controlled drug delivery with high hemostatic efficacy. Colloids Surf B Biointerfaces 2021;207:112013. 34339970 10.1016/j.colsurfb.2021.112013

[35] Malkov MI , Lee CT, Taylor CT. Regulation of the hypoxia-inducible factor (HIF) by pro-inflammatory cytokines. Cells 2021;10:2340. 34571989 10.3390/cells10092340 PMC8466990

[36] Ma J , Li Y, Yang X, Liu K, Zhang X, Zuo X, Ye R, Wang Z, Shi R, Meng Q, Chen X. Signaling pathways in vascular function and hypertension: molecular mechanisms and therapeutic interventions. Signal Transduct Target Ther 2023;8:168. 37080965 10.1038/s41392-023-01430-7 PMC10119183

[37] Hou J , Wang L, Long H, Wu H, Wu Q, Zhong T, Chen X, Zhou C, Guo T, Wang T. Hypoxia preconditioning promotes cardiac stem cell survival and cardiogenic differentiation in vitro involving activation of the HIF-1α/apelin/APJ axis. Stem Cell Res Ther 2017;8:215. 28962638 10.1186/s13287-017-0673-4 PMC5622481

[38] Wu J , Li S, Wang H, Qi Y, Tao S, Tang P, Liu D. High-yield BMSC-derived exosomes by the 3D culture system to enhance the skin wound repair. Regen Biomater 2025;12:rbaf022. 40309353 10.1093/rb/rbaf022 PMC12041419

[39] Zhu D , Hu Y, Kong X, Luo Y, Zhang Y, Wu Y, Tan J, Chen J, Xu T, Zhu L. Enhanced burn wound healing by controlled-release 3D ADMSC-derived exosome-loaded hyaluronan hydrogel. Regen Biomater 2024;11:rbae035. 38628545 10.1093/rb/rbae035 PMC11018541

[40] Su K , Wang C. Recent advances in the use of gelatin in biomedical research. Biotechnol Lett 2015;37:2139–45. 26160110 10.1007/s10529-015-1907-0

[41] Hou C , Zhang Y, Lv Z, Luan Y, Li J, Meng C, Liu K, Luo X, Chen L, Liu F. Macrophage exosomes modified by miR-365-2-5p promoted osteoblast osteogenic differentiation by targeting OLFML1. Regen Biomater 2024;11:rbae018. 38487712 10.1093/rb/rbae018 PMC10939467

[42] Chen J , Cheng X, Yu Z, Deng R, Cui R, Zhou J, Long H, Hu Y, Quan D, Bai Y. Sustained delivery of NT-3 and curcumin augments microenvironment modulation effects of decellularized spinal cord matrix hydrogel for spinal cord injury repair. Regen Biomater 2024;11:rbae039. 38746707 10.1093/rb/rbae039 PMC11090998

[43] Zhou X , Guo M, Wang Z, Wang Y, Zhang P. Rapid fabrication of biomimetic PLGA microsphere incorporated with natural porcine dermal aECM for bone regeneration. Regen Biomater 2024;11:rbae099. 39463918 10.1093/rb/rbae099 PMC11512121

[44] Sanches PHG , de Melo NC, Porcari AM, de Carvalho LM. Integrating molecular perspectives: strategies for comprehensive multi-omics integrative data analysis and machine learning applications in transcriptomics, proteomics, and metabolomics. Biology (Basel) 2024;13:848. 39596803 10.3390/biology13110848 PMC11592251

[45] Wörheide MA , Krumsiek J, Kastenmüller G, Arnold M. Multi-omics integration in biomedical research—a metabolomics-centric review. Anal Chim Acta 2021;1141:144–62. 33248648 10.1016/j.aca.2020.10.038 PMC7701361

